# Tick cysteine protease inhibitors suppress immune responses in mannan-induced psoriasis-like inflammation

**DOI:** 10.3389/fimmu.2024.1344878

**Published:** 2024-02-20

**Authors:** Huimei Wu, Mohamed Amine Jmel, Jinwei Chai, Maolin Tian, Xueqing Xu, Yuan Hui, Kutty Selva Nandakumar, Michail Kotsyfakis

**Affiliations:** ^1^ Guangzhou Medical Research Institute of Infectious Diseases, Department of Pharmacy, Guangzhou Eighth People’s Hospital, Guangzhou Medical University, Guangzhou, China; ^2^ Karolinska Institute United Medical Inflammation Center, School of Pharmaceutical Sciences, Southern Medical University, Guangzhou, China; ^3^ Institute of Parasitology, Biology Centre, Czech Academy of Sciences, České Budějovice, Czechia; ^4^ Guangdong Provincial Key Laboratory of New Drug Screening, School of Pharmaceutical Sciences, Southern Medical University, Guangzhou, China; ^5^ Department of Pulmonary and Critical Care Medicine, Zhujiang Hospital, Southern Medical University, Guangzhou, China; ^6^ Department of Endocrinology, Fifth Affiliated Hospital, Southern Medical University, Guangzhou, China; ^7^ Department of Environmental and Biosciences, School of Business, Innovation and Sustainability, Halmstad University, Halmstad, Sweden; ^8^ Institute of Molecular Biology and Biotechnology, Foundation for Research and Technology-Hellas, Heraklion, Crete, Greece

**Keywords:** autoimmune disease, psoriasis, tick, protease inhibitors, immune responses

## Abstract

Protease inhibitors regulate various biological processes and prevent host tissue/organ damage. Specific inhibition/regulation of proteases is clinically valuable for treating several diseases. Psoriasis affects the skin in the limbs and scalp of the body, and the contribution of cysteine and serine proteases to the development of skin inflammation is well documented. Cysteine protease inhibitors from ticks have high specificity, selectivity, and affinity to their target proteases and are efficient immunomodulators. However, their potential therapeutic effect on psoriasis pathogenesis remains to be determined. Therefore, we tested four tick cystatins (Sialostatin L, Sialostatin L2, Iristatin, and Mialostatin) in the recently developed, innate immunity-dependent mannan-induced psoriasis model. We explored the effects of protease inhibitors on clinical symptoms and histological features. In addition, the number and percentage of immune cells (dendritic cells, neutrophils, macrophages, and γδT cells) by flow cytometry, immunofluorescence/immunohistochemistry and, the expression of pro-inflammatory cytokines (TNF-a, IL-6, IL-22, IL-23, and IL-17 family) by qPCR were analyzed using skin, spleen, and lymph node samples. Tick protease inhibitors have significantly decreased psoriasis symptoms and disease manifestations but had differential effects on inflammatory responses and immune cell populations, suggesting different modes of action of these inhibitors on psoriasis-like inflammation. Thus, our study demonstrates, for the first time, the usefulness of tick-derived protease inhibitors for treating skin inflammation in patients.

## Introduction

Autoimmune diseases are comprised of a group of 70 different chronic diseases (1) characterized by inflammatory autoimmune responses to self-antigens and reported as a third leading cause of morbidity in the world (2). Psoriasis, one of the autoimmune diseases, is a chronic inflammatory skin disease with a complicated pathogenesis (3). Traditional drugs for treating psoriasis mainly include methotrexate, cyclosporine, retinoic acid, and glucocorticoids (4), but the disadvantages of slow action, limited efficacy, and adverse reactions limited their use. Biologics have drastically improved our ability to treat psoriasis and psoriatic arthritis over the last 20 years, which include inhibitors of cytokines or their receptors and enzyme inhibitors. Cytokine inhibitors are mostly recombinant monoclonal antibodies or receptor fusion proteins specific to inflammatory mediators, like TNF-α, IL-17, IL-12/IL-23 p40, and IL-23p19 (5). As an enzyme inhibitor, Apremilast inhibits phosphodiesterase 4 (PDE4), which in turn decreases the expression of pro-inflammatory cytokines TNF-α and IL-23, shows therapeutic efficacy in psoriasis, psoriatic arthropathies, and Behçet’s syndrome (6). Other enzyme inhibitors targeting Janus Kinase (JAK) (7) and tyrosine kinase 2 (TYK2) (8) show a significant therapeutic effect in the clinical and preclinical evaluations of psoriasis.

Tick saliva affects coagulation, complement activation, and immune response in terms of recruiting the immune cells, cytokine production, and cell maturation (9). It comprises a complex mixture of proteins and peptides (10, 11). Transcriptomic analysis of tick saliva shows the expression of various protease inhibitors (12, 13), including Kunitz-type inhibitors, cystatins, and serpins based on their structures and target proteases (10, 14). Several type 1 and 2 cystatins from tick saliva were described earlier. Sialostatin L, a type 1 cystatin identified in the saliva of hard tick *Ixodes scapularis*, modulates the cytokine production by lymphocytes, dendritic cells, and mast cells, causing impairment of T-cell expansion by inhibiting various (cathepsin C, L, S, V, X, and papain) enzymes (15). Another type 1 cystatin from the saliva of hard tick *Ixodes scapularis*, Sialostatin L2, suppresses the activities of cathepsins C, L, S, and V but not X (16), reduces the production of cytokines from the macrophages and inhibits caspase-1 maturation (17). As a type 2 cystatin, Iristatin inhibits proteolytic activities of cathepsins L and C, leading to decreased cytokine production from different T cells, mast cells, and nitric oxide production by macrophages. In addition, Iristatin inhibited OVA-induced CD4^+^ T-cell proliferation and leukocyte recruitment *in vivo*, indicating its versatility (18). Mialostatin, a cystatin from the midgut of *Ixodes ricinus*, inhibits several digestive cysteine-cathepsins, with a high-level potency observed against cathepsin L isoforms. Mialostatin has also effectively blocked *in vitro* proteolysis of blood proteins by midgut cysteine cathepsins (19).

Herein, we explored the effects of four cysteine protease inhibitors (Sialostatin L, Sialostatin L2, Iristatin, and Mialostatin) on mannan-induced psoriasis-like inflammation and evaluated the disease in terms of clinical and histological symptoms, infiltration of immune cells and expression of several pro-inflammatory cytokines.

## Materials and methods

### Mice

Eight to twelve-weeks-old BALB/c female mice purchased from Southern Medical University Animal Center were used and maintained in a pathogen-free animal house. Mice were placed in polystyrene cages in a climate-controlled environment with 12-h light/dark cycles and given food and water *ad libitum*. The animal studies were approved by the institutional review board of Southern Medical University (l2018183), Guangzhou, China, and performed according to the guidelines of the National Institutes of Health (NIH Publication No. 8023).

### Cloning, expression, and purification of the recombinant protease inhibitors

Sialostatin L, Sialostatin L2, Iristatin, and Mialostatin were expressed, purified and tested in presence of various proteases as described in detail elsewhere (16, 18–21). Briefly, the genes coding for the studied cystatins without a signal peptide but with an inserted ATG codon were cloned into a pET-17b vector and then transformed into *E.coli* strain BL21(DE3)pLysS. LB medium containing ampicillin (100 μg/ml) and chloramphenicol (34 μg/ml) was used for bacterial cultures until the optical density reached 0.8 at 600 nm. Protein expression was induced by adding 1 mM isopropyl 1-thio-β-D-galactopyranoside (IPTG). After 5 h, the cultures were harvested, and the inclusion bodies were recovered, washed, and dissolved in a solution containing 6 M guanidine hydrochloride, 20 mM Tris, and 10 mM DTT, pH 8. The refolded proteins were purified by HiLoad Superdex 200 26/60 gel filtration and ion exchange chromatographic methods. Endotoxin was removed by ARVYS proteins (Trumbull, Connecticut) using the Triton X-14 partitioning method (22) from all the recombinant protease inhibitors before using them in various *in vitro* and *in vivo* assays. Initial and final endotoxin concentrations were tested using Cambrex (Lonza) PyroGene Recombinant Factor C endotoxin detection system (Lonza Biologics) following manufacturer recommendations and the results are summarized in **Supplementary Table 1**. The toxicity of the tested cystatins was evaluated as reported in (23).

### Therapeutic effects of protease inhibitors on mannan-induced skin inflammation

To establish the mannan-induced skin inflammation (MISI) model, mice were shaved on the back skin one day before the treatment. Mannan (100 μl of 100 mg/ml stock solution, Sigma-Aldrich, USA) was mixed with incomplete Freund’s adjuvant (IFA, Sigma-Aldrich) at a 1:1 ratio and applied daily for three consecutive days, as described earlier (24). Induction of psoriasis in the inbred strains of mice than the classically used Imiquimod was reported to be relatively simple in inducing skin inflammation and found to be robust, economically more viable, and less harmful to the mice (25). The severity of the disease was assessed daily with a scoring system based on the development of scales, erythema, and skin thickness, like the human Psoriasis Area and Severity Index measurements with a scoring system having 0 to 4 scores (0, none; 1, mild; 2, moderate; 3, severe; 4, very severe) and a total score of 12. An increase in skin thickness was detected using an Ozaki digital caliper (Neill-Lavielle, Kentucky, USA). A detailed protocol was described elsewhere (26).

To investigate the effects of protease inhibitors on psoriasis-like inflammation, mice were randomly assigned to eight groups with at least six mice in each group as follows: PBS, mannan, negative control, Sialostatin L, Sialostatin L2, Iristatin or Mialostatin treated groups. Alpha-lactalbumin (Sigma-Aldrich) was used as a negative control. Different protease inhibitors were subcutaneously injected (4 mg/kg) individually from days 0 to 7. Skin, spleen, and the draining lymph node samples harvested on days 4 and 7 were used to evaluate the disease pathology, infiltrating immune cells, and the expression of cytokine genes.

### Histological, Immunohistochemical, and Immunofluorescent analysis

For hematoxylin and eosin (H&E) staining, skin samples were fixed in 4% formaldehyde and embedded in paraffin, and 6-8 μm sections were cut before the staining was performed. Histological sections were analyzed using LAS software version 4.9 (Leica, Germany) and a light microscope. Epidermal thickness was measured from the photomicrographs of skin sections after H&E staining by randomly selecting five regions using the Image Pro Plus software (Leeds Precision Instruments, USA). Baker’s scores were used to analyze the pathological severity of the skin (27), which has lesions in the stratum corneum, epidermis, and dermis. Neutrophils and macrophages were stained with biotin-rat anti-mouse Ly6G (1:200, BioLegend, USA) and F4/80 (1:100, BioLegend) antibodies, respectively, at 4°C overnight, followed by incubation with streptavidin-HRP antibodies (Yeasen, China) for 40 min. Before visualization, sections were developed with DAB (Vector Laboratories, California, USA) and counter-stained with hematoxylin (Phygene, China). For immunofluorescence staining, frozen samples were cut into 8 μm sections (Leica). At first, slices were permeabilized by acetone (Aladdin, China) and then incubated with the biotin-rat anti-mouse CD11c antibodies (1:100, BioLegend) overnight. Sections were treated with Alexa Fluor^®^ 488 conjugated streptavidin (1:800, Biyotimes, China) secondary antibodies at RT for 1 h. After washing with PBST (0.2% Tween-20 in PBS), the tissue slices were fixed with Vectashield® containing DAPI and used for visualization. Fluorescence pictures were acquired and analyzed using Nikon Laser Confocal Microscope and NIS Elements Viewer Imaging Software (Nikon).

### Flow cytometry

Each mouse lymph nodes and spleen were minced separately and passed through a 70 μm cell strainer to obtain single-cell suspensions. Single cells from the draining lymph nodes were used for the detection of innate immune cells and were stained with the following monoclonal antibodies: F4/80-PerCP-Cy 5.5, CD11c-PE, CD11b-APC, and Ly6G-FITC (BD Biosciences, USA). The γδT and Th17 cells from the spleen samples were detected with the following fluorescent antibodies: CD45-PerCP-Cy 5.5, γδT-PE, CD4-APC, and IL-17A-PE (BD Biosciences, USA). For the surface antigen staining, cells were incubated with the antibodies for 30 min at room temperature (RT) after washing the cells with PBS. To stain the intracellular cytokines, the cells were stimulated with a cell stimulation cocktail (eBiosicence) for 4 h. After that, the cells were stained for surface antigens and then fixed with BD Cytofix buffer, permeabilized by using Perm/Wash reagent (BD Biosciences) and then stained with anti-IL-17A antibodies. The cells were acquired using an LSR II Flow Cytometer (BD Biosciences), and the data were analyzed using the Flow Jo software version 7.0 (Tree Star, California, USA).

### RNA isolation and qRT-PCR

Total RNA was extracted from the skin using a Trizol reagent kit (Invitrogen, California, USA). Reverse transcription (RT) reactions were carried out with PrimeScript RT reagent Kit (ThermoFisher, USA), and a quantitative real-time polymerase chain reaction was performed using the SYBR Premix Ex Taq II (Takara biotech, Japan) in a LightCycler 96 thermocycler (Roche, Switzerland). The amplification program consists of 1 cycle of 95°C for 3 min followed by 45 cycles of 95°C for 5 s, 55°C for 5 s, and 72°C for 10 s and at the end, one cycle at 72°C for 5 min. VEGF, TGF-β, TNF-α, IL-6, IL-22, IL-23(P19), IL-17A, IL-17E, and IL-17F primers were purchased from Jierui (Shanghai, China), and β-actin was used as the internal control. The primers used to detect the expression of different genes are listed below *β-actin*, forward, 5’-ACCGTGAAAAGATGACCCAG-3’, and reverse 5’-GTACGACCAGAGGCATACAG-3’; *VEGF*, forward, 5’-GTCCTCTCCTTACCCCACCTCCT, and reverse 5’-CTCACACACACAGCCAAGTCTCCT-3’; *TGF-β*, forward, 5’-AGTGGAAGTGGTGCCTTTCAA-3’, and reverse, 5’-GTGAGACACCTCATCAG. GGTA-3’; *TNF-α*, forward, 5’-ACGCTCTTCTGTCTACTGAACT-3’, and reverse, 5’-ATCTGAGTGTGAGGGTCTGG-3’; *IL-6*, forward, 5’-GAGAAAAGAGTTGTGC AATGGC-3’, and reverse, 5’-CCAGTTTGGTAGCATCCATCAT-3’; *IL-22*, forward, 5’-CATGCAGGAGGTGGTACCTT-3’, and reverse, 5’-CAGACGCAAGCATTTCTC AG-3’; *IL-23-P19*, forward, 5’-AGCAACTTCACACCTCCCTAC-3’, and reverse, 5’-ACTGCTGACTAGAACTCAGGC-3’; *IL-17A*, forward, 5’-CCCCTAAGAAACCC CCACG-3’, and reverse, 5’-TAAAGTCCACAGAAAAACAAACACG-3’; *IL-17E*, forward, 5’-ACAGGGACTTGAATCGGGTC-3’, and reverse, 5’-TGGTAAAGTGGG ACGGAGTTG-3’; *IL-17F*, forward, 5’-GTCAGGAAGACAGCACCA-3’, and reverse 5’-AGCCAACTTTTAGGAGCA-3’;*IL-10*,forward,5′-GGCCTTCCCTACTTCACAA G-3′,and reverse 5′-GGCCTTCCCTACTTCACAAG-3′; *IL-4*,5′-GTCATCCTGCTC TTCTTTCTCG-3′, and reverse 5′-TTGGCACATCCATCTCCGT-3′.

### Statistical analysis

Data were analyzed using GraphPad Prism software version 5.0. For standard data sets, data were shown as mean ± SEM, and an unpaired two-tailed Student’s t-test was used for statistical analyses. For multiple groups, a one-way analysis of variance was applied. In the qPCR analysis, an average value for the expression of a particular gene from the mannan-treated group (Man) was given the value “1” (standard) and used for comparison with other groups. Probability values < 0.05 were considered significant at a 95% confidence interval.

## Results

### Tick protease inhibitors and their structures

This study explores the therapeutic effects of four tick cystatins previously reported for their various immunomodulatory activities and specificity towards key cysteine proteases involved in different pathways related to immunity, inflammation, and homeostasis. Sialostatin L and Sialostatin L2, two cystatins isolated from the salivary glands of *Ixodes scapularis* (15–17, 23, 28–31), Iristatin, a salivary gland cystatin and Mialostatin, a midgut cystatin were isolated from *Ixodes ricinus* (18, 19, 21). **Table 1** and **Supplementary Figure 1** show the protease inhibitors, their primary targets, and published structures.

**Table 1:** Summary of the tested tick protease inhibitors.

Protease inhibitor	Sources	Inhibited proteases	Effects on immune systems
Sialostatin L	Salivary glands of *Ixodes scapularis*	Cathepsins L, S, C, and X, Papain	Modulates cytokine production by immune cells and impairs T-cell proliferation (31)
Sialostatin L2	Salivary glands of *Ixodes scapularis*	Cathepsins L, S and C Papain (20)	Reduces cytokine(s) production from macrophages and inhibits caspase-1 maturation (20)
Iristatin	Salivary glands of *Ixodes ricinus*	Cathepsins L and C	Decreases cytokine(s) and nitric oxide production from immune cells, inhibits T-cell proliferation and leukocyte recruitment (18).
Mialostatin	Midgut of *Ixodes ricinus*	Human cathepsins L, C, S, B, K and H	Blocks proteolysis of blood proteins (19)

In addition to their structure and functions, we report the inhibitory potency of the studied cystatins on various proteases involved in key immune pathways such as cathepsins L, S, and C (**Supplementary Table 2**). Amino acid sequences of the cystatins used in this study are given in **Supplementary Table 3**.

### Tick protease inhibitors ameliorate mannan-induced skin inflammation

Toxicity analysis of the used tick protease inhibitors on BMDMs did not show any toxic effects (**Supplementary Figure 2**). To explore the effects of these inhibitors on the pathogenesis of psoriasis-like inflammation, we treated psoriatic mice by injecting an individual inhibitor. We monitored disease severity, epidermal thickness, and pathological manifestations at the microscopic level and also evaluated the expression of specific growth factors, like VEGF and TGF-β.

**Figure 1:**
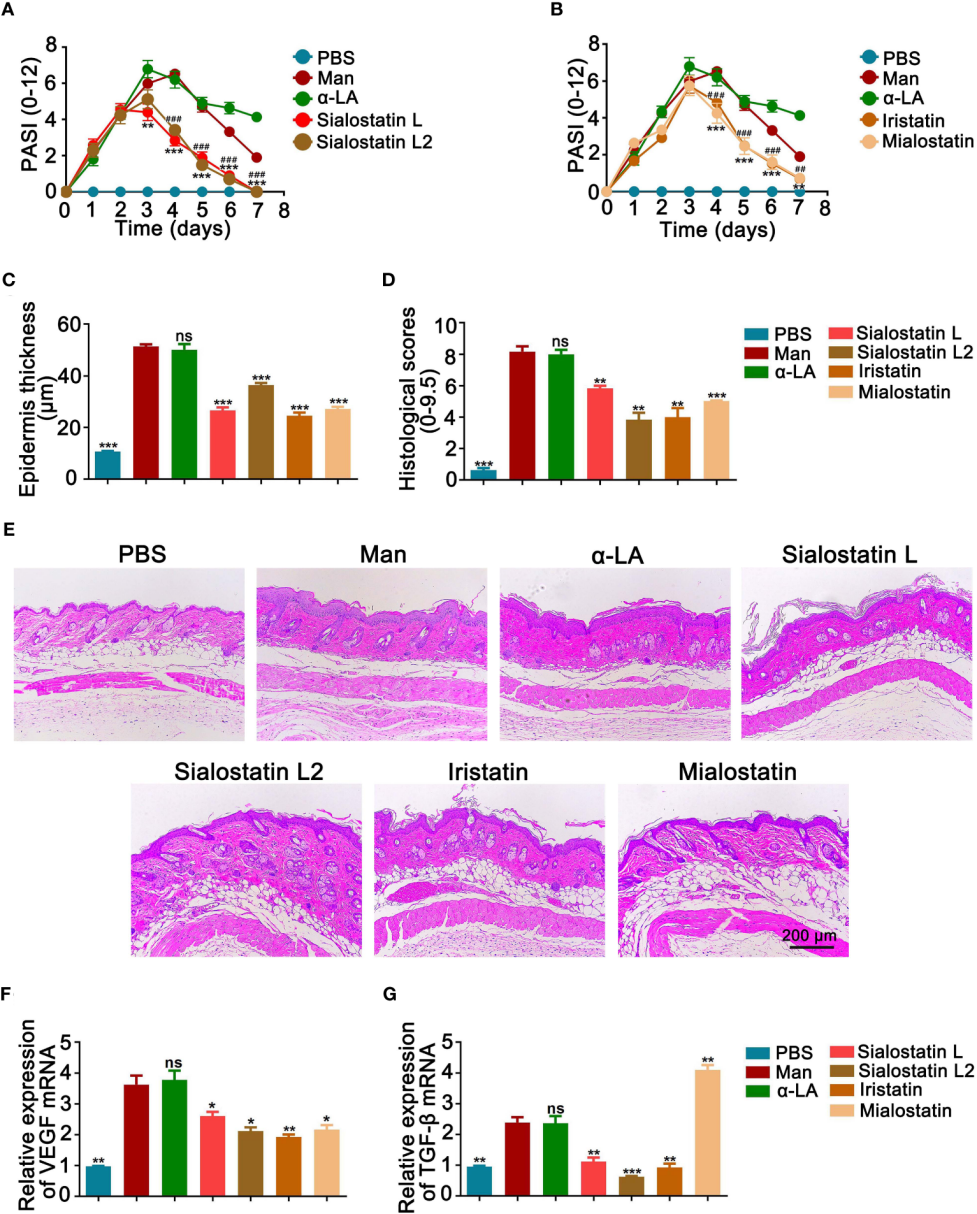
Tick protease inhibitors decreased psoriasis severity and microscopical disease manifestations. Effect of Sialostatin L, L2 **(A)** Iristatin or Mialostatin **(B)** treatment in MISI. **(C, D)** Statistical results showing the epidermal thickness and histological scores of psoriatic skins from sialostatin L, Sialostatin L2, Iristatin, or Mialostatin-treated mice (n = 12/group). **(E)** Representative pictures of H&E-stained skin sections (n = 5/group). The scale bar is 200 μm. **(F, G)** Expression of VEGF and TGF-β in psoriatic skin (n = 5/group). Man, Mannan; α-LA, alpha-lactalbumin, negative control. Results are shown from a representative experiment. All mice developed psoriasis-like inflammation. Statistical analysis was performed using an unpaired t-test; ns, not significant; *, p < 0.05; **, p < 0.01; ***, p < 0.001. #, p < 0.05; ##, p < 0.01; ###, p < 0.001. *** in Panel A was used to show the difference between mannan and Sialostatin L group. ### in Panel A showed significance between mannan and Sialostatin L2 group, and indicates p < 0.001.

Cysteine protease inhibitors treatment significantly decreased psoriasis area and severity index (PASI) compared to the untreated group (**Figures 1A, B**), with a more prominent effect observed from day 3 onwards in the case of Sialostatin L and Sialostatin L2. In contrast, the effect of Iristatin and Mialostatin was observed a day later as they significantly improved PASI from day 4 onwards. On the other hand, a significant decrease in the epidermal thickness and histological scores was observed on day 4 (**Figures 1C, D**). The highest reduction in the epidermal thickness was observed for Sialostatin L (26.6 µm), Iristatin (24.5 µm), and Mialostatin (27.1 µm). Sialostatin L2 had a lower yet significant effect (36.4 µm), as shown in **Figures 1C, E**.

Based on the crucial role of TGF-β1 in inducing angiogenesis in the skin through the VEGF-mediated apoptosis (32), we analyzed the expression patterns of VEGF and TGF-β in the inflamed areas of the treated and untreated skin. We found a significantly lower level of VEGF mRNA expression at the peak of psoriasis after treatment with the tick cystatins, with a more significant effect observed with Iristatin treatment (**Figure 1F**). On the other hand, all the salivary protease inhibitors, except Mialostatin, down-regulated TGF-β mRNA expression in the inflamed skin. The midgut protease inhibitor, Mialostatin, significantly increased its expression (**Figure 1G**), showing that the inhibitory effects of the protease inhibitors on TGF-β expression were dependent on the origin of inhibitors in the ticks.

### Modulation of innate immune cells contributes to the improvement of PASI

**Figure 2:**
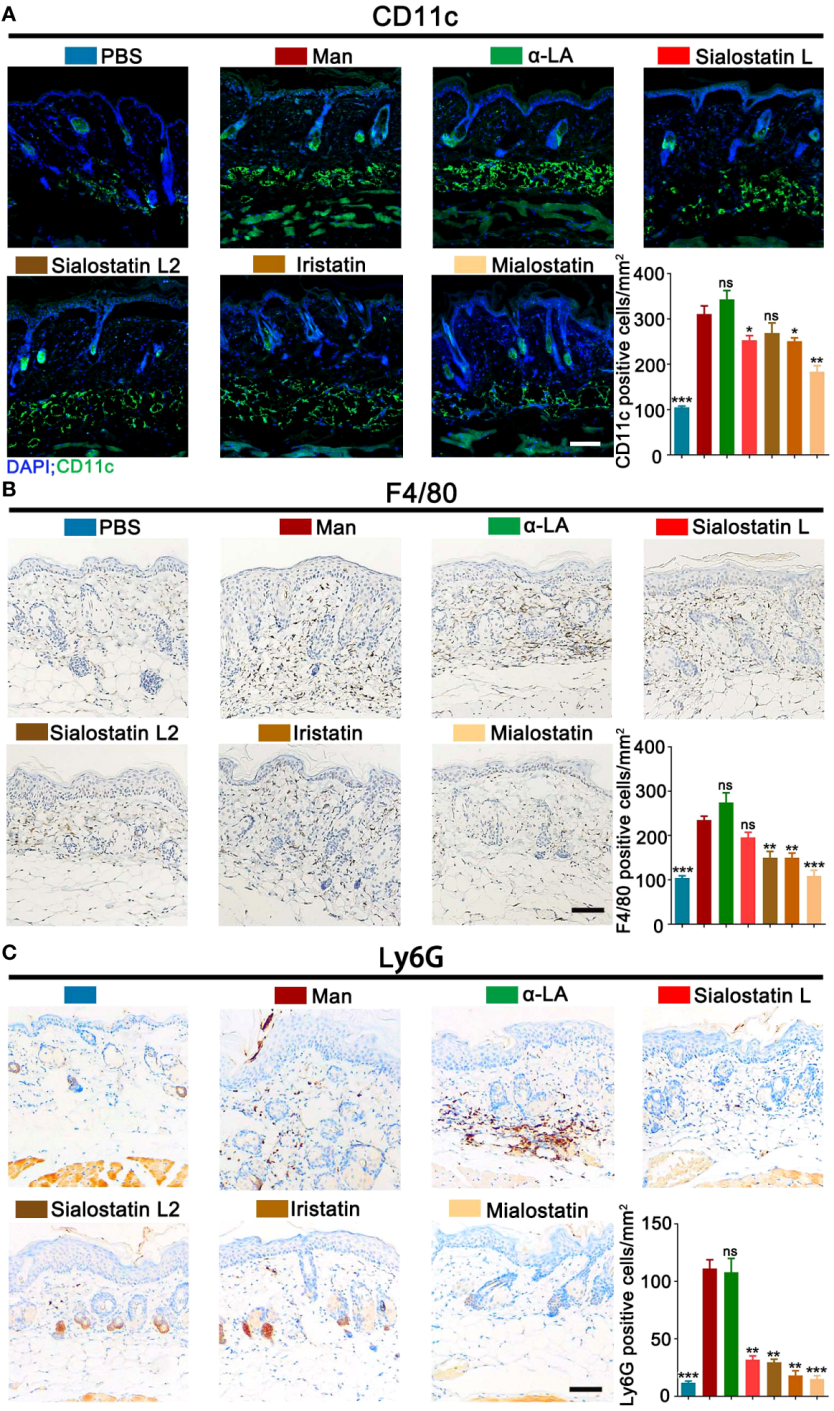
Tick protease inhibitors improved mannan-induced skin inflammation by modulating infiltrated immune cells in the skin lesions. **(A)** Representative pictures of immunofluorescence staining and statistical results show CD11c^+^ cells (green) expression in MISI after Sialostatin L, Sialostatin L2, Iristatin, or Mialostatin treatment (n = 3/group). Nuclei were counterstained with DAPI (blue). **(B, C)** Immunohistochemistry staining and statistical results show F4/80^+^ and Ly6G^+^ cell expression in the tick protease inhibitors treated mice (n = 3/group). The scale bar is 100 μm. Positive cells in each mm^2^ were calculated under optical and confocal microscopy. Man, mannan; α-LA, alpha-lactalbumin, negative control. Results are from a representative experiment. All mice developed psoriasis-like inflammation. Statistical analysis was performed using an unpaired t-test; ns, not significant; *, p < 0.05; **, p < 0.01; ***, p < 0.001.

The infiltration of immune cells to the skin inflammation area was evaluated by measuring the percentage of innate immune cells. CD11b^+^CD11c^+^ (Dendritic cells), CD11b^+^F4/80^+^ (Macrophages), and CD11b^+^Ly6G^+^ (Neutrophils) cells were detected in the different groups to assess the effect of the tick salivary cystatins on these cells at the skin lesional area. We observed variable effects depending on the studied immune cell and the injected protease inhibitor (**Figures 2A–C**). The most significant effect on dendritic cell percentage in the lesional area was observed with Mialostatin, followed by a similar effect after Iristatin or Sialostatin L treatments. However, sialostatin L2 didn’t affect the percentage of DCs present in the skin lesions (**Figure 2A**). On the other hand, macrophage infiltration to the inflammation site was most prominently impaired by Mialostatin, followed by Iristatin and Sialostatin L2. Meanwhile, Sialostatin L didn’t significantly inhibit macrophage migration (**Figure 2B**). Finally, neutrophil infiltration in the skin lesions was altered by all four tested protease inhibitors, with the most distinct effect observed with Mialostatin (**Figure 2C**).

**Figure 3:**
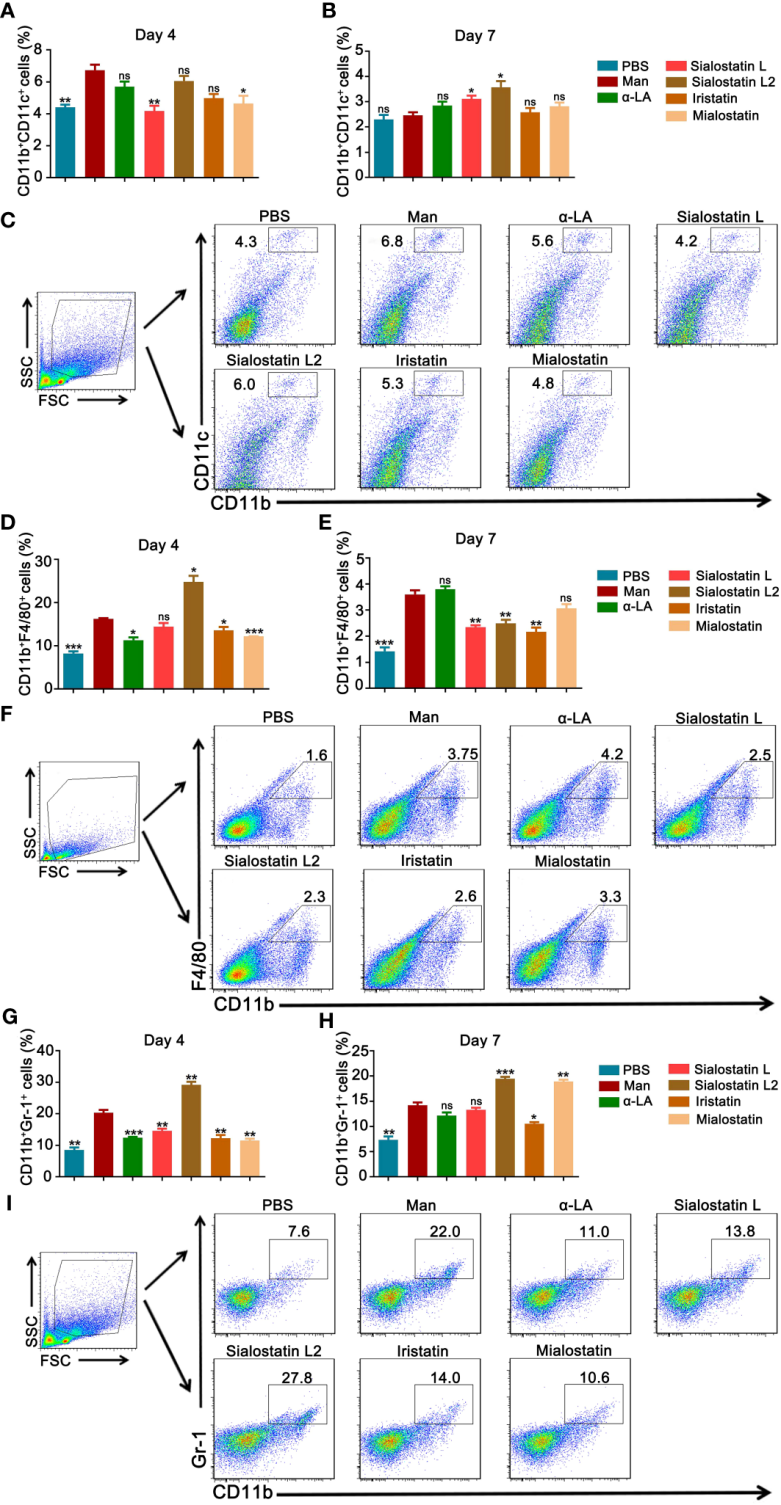
Inhibiting innate immune cells from the spleen improves MISI by tick protease inhibitors. **(A, B)** Statistical results showing the effects of Sialostatin L, Sialostatin L2, Iristatin, and Mialostatin on dendritic cells (CD11b^+^CD11c^+^) at days 4 and 7 (n = 5/group). **(C)** Gating strategy and representative pictures showing CD11b^+^CD11c^+^ cells in the spleen at day 4 in the tick protease inhibitors treated mice (n = 5/group). **(D, E)** Effects of Sialostatin L, Sialostatin L2, Iristatin or Mialostatin on macrophages (CD11b^+^F4/80^+^) expression at day 4 and 7 (n = 5/group). **(F)** Representative plots of spleen cells from mice in MISI at day 7 that were treated with Sialostatin L, Sialostatin L2, Iristatin, or Mialostatin (n = 5/group). **(G, H)** Effects of Sialostatin L, Sialostatin L2, Iristatin, or Mialostatin on neutrophils (CD11b^+^Ly6G^+^ cells) expression in the spleen cells at day 4 and 7 were analyzed by flow cytometry (n = 5/group). **(I)** Representative pictures showing macrophage staining (CD11b^+^ cells) within the spleen cells from protease inhibitors treated mice (n = 5/group). Man, mannan; α-LA, alpha-lactalbumin, negative control. Results are shown from a representative experiment. All mice developed psoriasis-like inflammation. Statistical analysis was performed using an unpaired t-test; ns, not significant; *, p < 0.05; **, p < 0.01; ***, p < 0.001.

As tick-derived cystatins were repeatedly reported for their immunomodulatory and immunosuppressive activities, we analyzed the effects of all four protease inhibitors on the expression of innate immune cells in the secondary lymphoid organs, and we detected their expression in the spleen at days 4 and 7 using flow cytometry (**Figures 3A–I**).

Dendritic cells significantly contribute to psoriasis development during the initiation and maintenance phases (33). The tested cystatins affected CD11b^+^CD11c^+^ cells differently (**Figures 3A–C**). On day 4, we observed a significant inhibitory effect of Sialostatin L and a moderate effect with Mialostatin while Sialostatin L2 and Iristatin had no effects (**Figure 3A**). At the declining phase of psoriasis (Day 7), Sialostatin L still had a significant effect on CD11b^+^CD11c^+^ cells, albeit at a lower level. At the same time, Mialostatin lost its immunomodulatory effects on these cells (**Figure 3B**). On the other hand, dendritic cell overexpression was observed in the presence of Sialostatin L2, while Iristatin was still ineffective from days 4 to 7. The observed inhibition of dendritic cells suggests that the proteases secreted by these cells could be a primary target for the protease inhibitors Sialostatin L and Mialostatin.

The indispensable role of skin macrophages in developing psoriasis-like inflammation of K14-Cre-IKK2^fl/fl^ mice was documented earlier (34). Similarly, depletion of monocytes/macrophages using clodronate liposomes reduced mannan-induced joint and skin inflammation in B10Q*.Ncf1* ^m1j/m1j^ mice (35). In this study, CD11b^+^F4/80^+^ cells (**Figures 3D–F**) were mainly inhibited by Mialostatin and Iristatin at day 4, while Sialostatin L2 significantly increased their expression (**Figure 3D**). At the end of psoriasis (Day 7), we observed a decrease in the number of macrophages after treatment with Sialostatin L, Sialostatin L2, and Iristatin. At the same time, Mialostatin had no effect (**Figure 3E**).

The contribution of neutrophils in mannan-induced joint and skin inflammation in B10Q*.Ncf1* ^m1j/m1j^ mice was documented earlier by depleting them using anti-Ly6G antibodies (35). Here, we observed a significant inhibition of the percentage of neutrophils in the spleen on day 4 (**Figures 3G–I**) by Sialostatin L, Mialostatin and Iristatin. However, on day 7, only Iristatin had a significant inhibitory effect. The percentage of neutrophils increased in the Mialostatin-treated mice on day 7, and there was no significant difference between untreated and Silaostatin L-treated mice, suggesting a requirement for a higher concentration of these inhibitors for treatment. In contrast, Sialostatin L2 had a significant activating effect on neutrophil expression both on days 4 and 7, which warrants further experiments to decipher this anomaly.

### The γδT and Th17 cells contribute to the therapeutic effect

**Figure 4:**
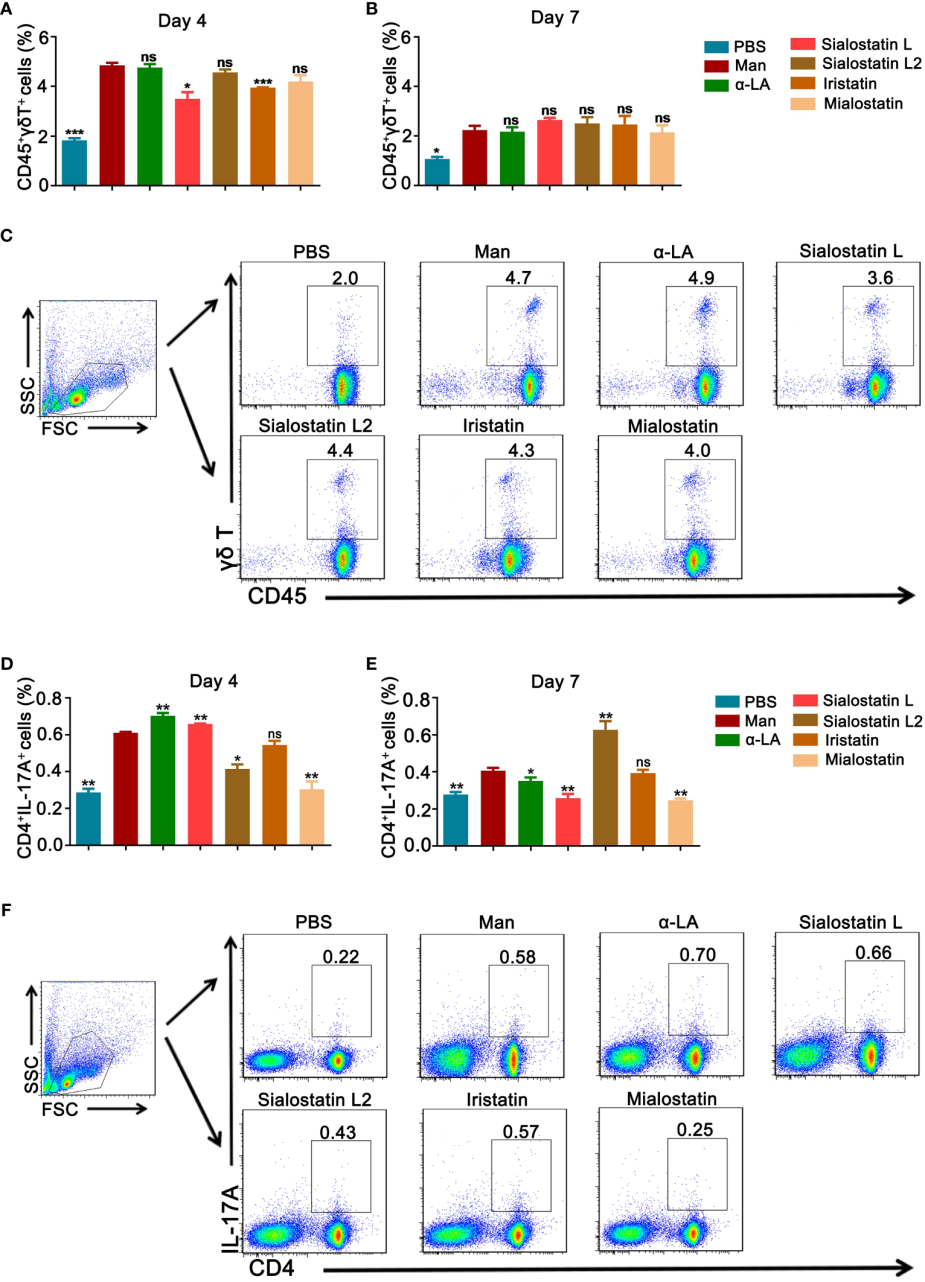
Effect of tick protease inhibitors on γδ T and Th17 cells at the peak and end of psoriasis. **(A, B)** Statistical analysis on the effect of Sialostatin L, Sialostatin L2, Iristatin, or Mialostatin on γδ T cells from draining lymph nodes at the peak (day 4) as well as the end (day 7) of psoriasis (n = 5/group). **(C)** Gating strategy and representative blots showing γδ T cells (CD45^+^γδ T^+^) expression at day 4 in the tick protease inhibitors treated mice (n = 5/group). **(D, E)** The spleen cells’ frequency is depicted as a fraction of total Th17 cells at days 4 and 7 after Sialostatin L, Sialostatin L2, Iristatin, or Mialostatin treatment in MISI (n = 5/group). **(F)** Representative pictures show CD4^+^ IL-17A^+^ positive cells from the spleen at day 4 in MISI after tick protease inhibitor treatment (n = 5/group). Man, mannan; α-LA, alpha-lactalbumin, negative control. Results are shown from a representative experiment. All mice developed psoriasis-like inflammation. Statistical analysis was performed using an unpaired t-test; n ns, not significant; *, p < 0.05; **, p < 0.01; ***, p < 0.001.

Psoriasis development highly depends on the γδT and Th17 cell axis (36, 37). Thus, we evaluated the effect of the protease inhibitors on these cells to understand their implication and effects on the development of Psoriasis. Only Sialostatin L and Iristatin decreased CD45^+^γδT^+^ cells in the draining lymph nodes at day 4 (**Figures 4A, C**). However, none of the protease inhibitors significantly affected these cells on day 7 (**Figure 4B**).

On the other hand, in the draining lymph nodes, only Mialostatin and Sialostatin L2 significantly inhibited the percentage of Th17 cells on day 4 (**Figures 4D, F**); however, we observed an increase in the presence of Sialostatin L. On day 7 (**Figure 4E**), Mialostatin inhibited the percentage of Th17 cells in addition to Sialostatin L, while Iristatin remained ineffective. An increase in Th17 cells was also observed in the case of Sialostatin L2.

### Tick protease inhibitors ameliorate mannan induced skin inflammation by altering the expression of pro- and anti-inflammatory cytokine genes

**Figure 5:**
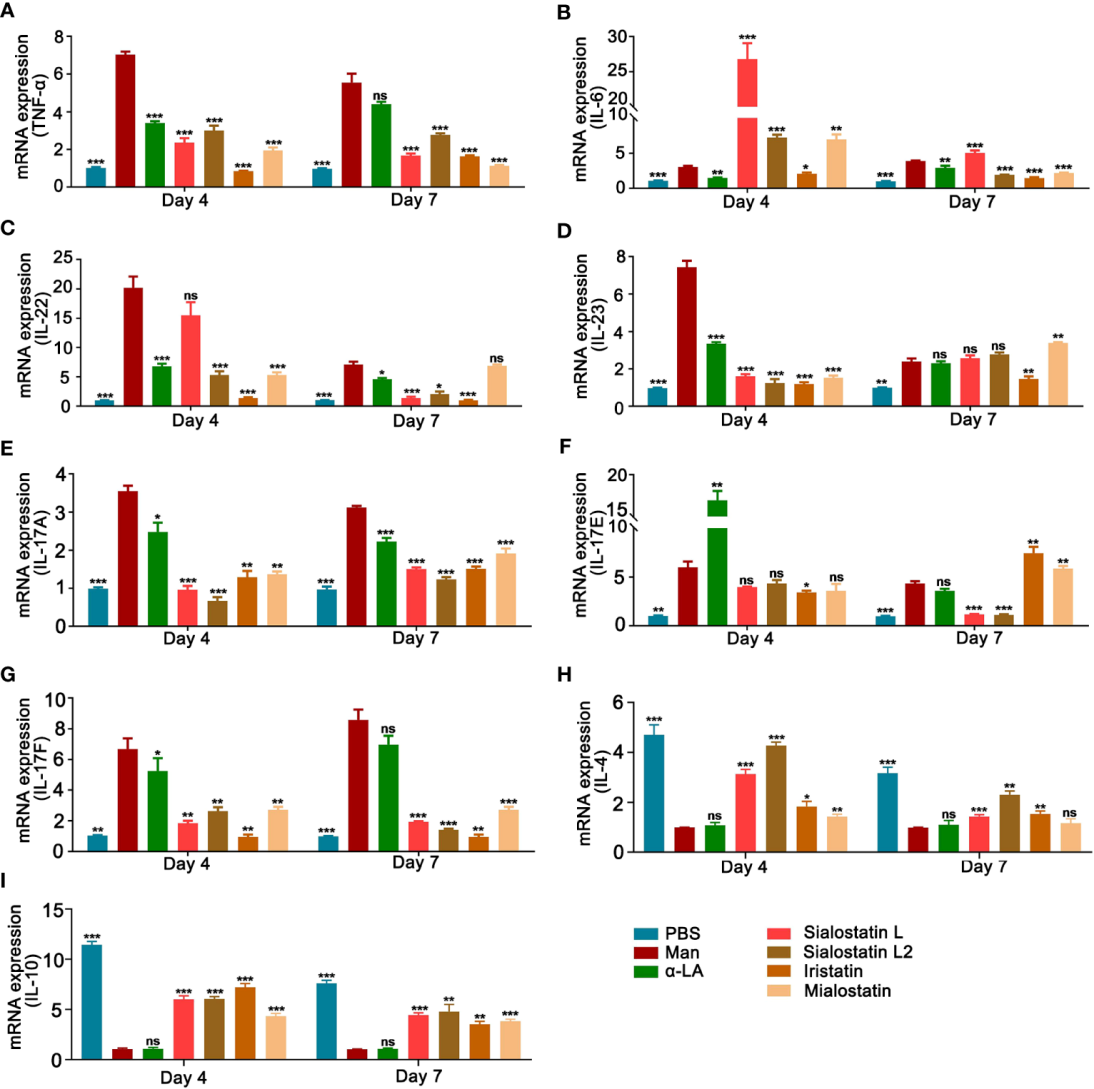
Tick protease inhibitors decreased mannan-induced skin inflammation by modulating the expression of pro-inflammatory cytokines. Statistical analysis showing the effects of Sialostatin L, Sialostatin L2, Iristatin, or Mialostatin on the mRNA expression of **(A)** TNF-α, **(B)** IL-6, **(C)** IL-22, **(D)** IL-23 **(E)** IL-17A, **(F)** IL-17E, **(G)** IL-17F, **(H)** IL-4 and **(I)** IL-10 in the skin lesions at the peak (day 4) as well as the end (day 7) of mannan induced skin inflammation after Sialostatin L, Sialostatin L2, Iristatin or Mialostatin treatment (n = 5/group). **(A–G)** PBS group was normalized to 1, and **(H, I)** mannan group was normalized to 1 for showing the differences between each group. Man, mannan; α-LA, alpha-lactalbumin, negative control. All mice developed psoriasis-like inflammation. Statistical analysis was performed using an unpaired t-test; ns, not significant; *, p < 0.05; **, p < 0.01; ***, p < 0.001.

The abnormal proliferation of epidermal keratinocytes during psoriasis is mainly due to the pro-inflammatory cytokines secreted by keratinocytes and resident immune cells such as dendritic cells, T cells, and other innate immune cells (38, 39). Here, we investigated the expression of psoriasis-related cytokines (TNF-α, IL-6, IL-22, IL-23, IL-17A, IL-17E, IL-17F, IL-4 and IL-10) in the inflamed skin after mannan exposure at the peak (day 4) and the end (day 7) of psoriasis with/without inhibitor(s) treatment. Although IL-1β and TNF-α significantly induced IL-17A production from mannan-activated skin cells, only TNF-α but not IL-1β neutralization had a significant effect on joint and skin inflammation induced by an intraperitoneal injection of mannan in B10Q*.Ncf1* ^m1j/m1j^ mice (35). In this study, all the inhibitors, mainly Iristatin, significantly decreased TNF-α expression during psoriasis development (**Figure 5A**).

IL-6, a key cytokine secreted by macrophages that promotes keratinocyte proliferation, showed an unusual expression pattern. Except for Iristatin, all the other tested cystatins increased the expression of IL-6 at the peak of psoriasis (Day 4). At the end of psoriasis (Day 7), IL-6 expression increased with the Sialostatin L treatment, while Sialostatin L2, Iristatin, and Mialostatin significantly decreased its expression (**Figure 5B**).

We also analyzed the expression of IL-22, a cytokine produced in response to IL-6 and TNF-α, which has a crucial function in developing dermal inflammation and epidermal acanthosis (40). At the peak of MISI (day 4), Sialostatin L2, Iristatin, and Mialostatin decreased IL-22 mRNA expression, while Sialostatin L showed no significant inhibition (**Figure 5C**). On day 7, IL-22 was inhibited by all the cystatins except Mialostatin, suggesting different inhibitory time kinetics for various cystatins.

Among the IL-17 family upstream cytokines, IL-23 is expressed by the activated macrophages and dendritic cells and acts as a critical cytokine in psoriasis pathogenesis. IL-23 mRNA level in the inflamed skin was significantly reduced after subcutaneous injection of Sialostatin L, Sialostatin L2, Iristatin, or Mialostatin at the peak of psoriasis (**Figure 5D**), suggesting a direct connection between the action of tick protease inhibitors on the proteases secreted by the antigen-presenting cells. At the end of psoriasis (Day 7), only Iristatin kept its inhibitory effect on IL-23 expression, while Mialostatin significantly increased it, while sialostatin L and Sialostatin L2 didn’t show any effect.

A previous report showed that the Th17 family of cytokines (IL-17A and IL-17F) secreted by skin contained infiltrating γδT cells and RORγt^+^ innate lymphocytes, which promoted the initiation of IMQ-induced psoriasis (41). Subcutaneous injection of Sialostatin L, Sialostatin L2, Iristatin, or Mialostatin in the psoriatic mice decreased IL-17A and IL-17F mRNA expression at days 4 and 7 (**Figures 5E, G**).

On the other hand, when we evaluated the expression of IL-17E (IL-25) cytokine that contributes to the Th2 type of immune responses (42), the cystatin(s) effect was unclear and needed further clarification. Only Iristatin inhibited IL-17E expression at the peak of psoriasis. By day 7, we observed an inhibition after the injection of Sialostatin L and Sialostatin L2, while an increase in the expression was observed in the case of Iristatin and Mialostatin. (**Figure 5F**).

Interleukin-10 (IL-10) is a member of cytokine family produced by monocytes, Th2 cells and keratinocytes (43). Earlier studies documented the low-level expression of IL-10 in psoriasis and its reversal after the conventional anti-psoriatic therapies (44). The therapeutic response of IL-10 in psoriasis is associated with suppression of cutaneous inflammation, downregulation of the IL-8/CXCR2 pathway, and normalization of keratinocyte maturation (45). Th2 cells, basophils, mast cells, NK T cells, and type II innate lymphoid cells produce IL-4 (46) and IL-4 therapy in psoriasis patients induced Th2 responses and improved psoriasis (47). Our results show that there is a significant decrease in the expression of IL-10 in MISI, whereas Sialostatin L, Sialostatin L2, Iristatin, and Mialostatin reversed their expression during the inflammatory and declining phase of psoriasis on days 4 and 7. Except for Mialostatin, which has a significant effect on the expression of IL-4 at day 4, other inhibitors significantly reversed the IL-4 expression on days 4 and 7 (**Figures 5H, I**).

Of note, alpha-lactalbumin (α-LA) was used as a protein control in all the experiments. Hence, this protein could have its own effects in the animals on the expression of cytokine genes, activation of immune cells in the skin, and immune cells derived from the spleen, which explains the variations between the untreated and the α-LA treated groups.

## Discussion

Cysteine proteases are indispensable in various physiological processes and contribute to the development of several diseases. The role of these proteases in the processing of antigens for presentation, maturation of MHC class II molecules, apoptosis, autophagy, extracellular matrix remodeling, activation of granzymes in T cells, coagulation, cytokines and pro-hormones maturation, breakdown of intracellular proteins, cell growth, and differentiation are well known (48, 49). Therefore, cysteine proteases are potential drug targets in treating the underlying pathological processes in tissue degeneration and inflammation. Therapeutic targeting of these proteases could alter specific changes in cell functions. In this context, the relevance of tick protease inhibitors as therapeutics is increasingly highlighted as more and more proteins target host hemostasis, inflammation, and immunity with a unique and precise mechanism of action (50). In this work, we investigated the effect of a panel of tick cysteine protease inhibitors on the previously established psoriasis model in mice (24) to demonstrate their potential for developing pharmacological applications.

Psoriasis, an inflammatory skin disease affecting over 60 million people worldwide with an enormous negative effect on their psychosocial well-being (5), remains one of the significant challenges for modern medicine as no cure has yet been discovered to treat this disease. Only a few treatments aim to minimize the patients’ physical burden, which usually reaches extreme conditions such as depression (51) or suicide (52, 53).

The biologics used for the treatment of moderate and severe psoriasis are mainly receptor fusion proteins or monoclonal antibodies, and they can be divided into four classes, namely anti-TNFα, anti-IL17, anti-IL-23p40 (also known as anti-IL-12p40), and anti-IL-23p19 (5, 7, 38). Except for Infliximab, an anti-TNFα biologic administrated by an intravenous infusion, all the biologics targeting psoriasis are delivered by subcutaneous injections (5) as proceeded similarly in this work using the tick protease inhibitors. As the number of FDA-approved biologics keeps increasing, a permanent need for new therapeutics with increased specificity and lower side effects is demanded in parallel with the increasing knowledge about disease pathogenesis.

Since the biologics used for psoriasis treatments are mainly immunomodulators (54), tick-derived protease inhibitors can play a pivotal role by targeting the central cytokines involved in the inflammatory pathogenesis of the disease (42, 55, 56). Changes in the balance between the proteolytic function of proteases and their regulators (natural inhibitors) present in the skin can result in inflammation, leading to clinical signs of redness, scaling, and itching. Previously, cathepsins and cysteine proteases were reported to have an essential function in the development of psoriasis (49, 57, 58). Here, we tested four cysteine protease inhibitors (cystatins) that showed promising immunomodulatory activities and pluripotent effects on immune cells and targets. The tested inhibitors are Sialostatin L and Sialostatin L2, two cystatins from the salivary glands of *Ixodes scapularis* (15–17, 23, 28–31), Iristatin, a cystatin from the salivary glands of *Ixodes ricinus* (18, 21) and finally Mialostatin, a midgut cystatin from *Ixodes ricinus* (19).

Following the subcutaneous injection of the protease inhibitors, we observed a significant decrease in the macroscopical and microscopical inflammation induced by mannan in the skin. However, the immune responses targeted were different for each protease inhibitor. Both Sialostatin L and Iristatin showed an overall therapeutic capacity by decreasing the percentage of innate immune cells, T cells, and pro-inflammatory cytokine expression in the skin lesions and secondary lymphoid organs. On the other hand, Sialostatin L2 mainly affected the immune cells and cytokines from the inflamed skin but not the draining lymph node. Mialostatin modulated the immune responses by reducing the percentage of macrophages, dendritic cells, neutrophils, Th17 cells and cytokines.

Tick salivary glands and midguts are recognized as a rich source of pharmaco-active molecules (50), which contains a rich cocktail of proteins with a remarkable binding affinity, avidity, and selectivity for their targets in various host defense systems (10). Sialostatin L, a cysteine protease inhibitor from tick saliva, shows multifunctional effects on the immune system, which not only inhibits the percentage of CD4^+^ cells (31) but also reduces the production of IL-9 from Th9 cells in an OVA-induced experimental asthma (28). In this work, Sialostatin L decreased mannan-induced experimental psoriasis via a unique immunomodulatory effect by inhibiting the proliferation of γδ T cells and Th17 cells. In a previous study, Sialostatin L inhibited LPS-induced maturation of dendritic cells by affecting cathepsin S activity *in vivo* (15). Indeed, our result confirmed that Sialostatin L inhibits the proliferation of dendritic cells and other innate immune cells (macrophages and neutrophils) expression in MISI. In another study on Sialostatin L, the authors showed that this cystatin restrained IL-9, IL-1β, and IRF4 secretion from mast cells, whereas degranulation and IL-6 expression were unaffected (23). Effectively, Sialostatin L didn’t show any significant inhibition of IL-6, although psoriasis-related pro-inflammatory cytokines (TNF-α, IL-22, IL-23/IL-17) expression was reduced.

Sialostatin L2, the second salivary cystatin from *Ixodes scapularis*, binds to Annexin A2 and impairs NLRC4 inflammasome formation following macrophage infection (30). It affects IFN-β mediated immune reactions and tick-borne encephalitis (TBE) virus replications in mouse dendritic cells (29). When Sialostatin L2 was subcutaneously injected, it reduced mannan-induced psoriasis by affecting the number of macrophages in lesional skin and spleen and dendritic cells in the skin. Interestingly, it also decreased neutrophils and the expression of IL-23/IL-17 axis cytokines in the skin lesions and Th17 cells expression in the lymph nodes. This immunomodulation pattern allowed Sialostatin L2 to extenuate psoriasis by reducing the number of innate immune and Th17 cells, and the expression of pro-inflammatory cytokines.

Compared to Sialostatin L and Sialostatin L2, fewer reports dealt with the other tested protease inhibitors, namely Mialostatin and Iristatin. The latter is also a pluripotential cystatin, mainly affecting the adaptive immune system by diminishing IL-2, IL-4, IL-9, and IFN-γ production by different T-cell populations. At the same time, it also affects cytokines and nitric oxide secretion from the activated macrophages (18). Following the mannan application, Iristatin didn’t affect the Th17 cells, which aligns with the previous reports of Kotal et al. (18). Also, in accordance with the latter reference, Iristatin inhibited macrophages and neutrophils in the lesional skin and spleen. In addition, the decrease in psoriasis severity after applying Iristatin is mainly explained by the critical inhibition of the IL-6/IL-23/IL-17 axis cytokines, which are the main contributors to the disease pathogenesis (38).

The only midgut protein from *Ixodes ricinus* tested in this work, Mialostatin, was reported to inhibit some of the hard tick digestive cysteine cathepsins, with the greatest potency observed against cathepsin L isoforms. Although the effect of Mialostatin on various immune responses remained unexplored, this cystatin effectively blocked *in vitro* proteolysis of blood proteins (19). It reduced erythema (data not shown) and mRNA expression of VEGF in the lesional skin after mannan stimulation, which can be correlated with its inhibition on proteolysis of blood proteins. Mialostatin also showed interesting anti-inflammatory and immunosuppressive effects in MISI by reducing the percentage of innate immune cells and pro-inflammatory cytokines in the psoriasis skin, and the Th17 cells in draining lymph nodes. The subcutaneous injection of Mialostatin showed promising therapeutic effects in MISI. It was the most effective among the tested cystatins in decreasing the number of macrophages, dendritic cells, and neutrophils to the skin lesions.

Neutralizing the different cytokines involved in psoriasis remains a prominent approach of high clinical relevance in treating the disease (56). At the same time, drugs targeting several IL-17 are highly in demand, with several drugs being tested in advanced clinical stages (59). Bimekizumab represents a good example, targeting IL-17A and IL-17F (55). Here, we report tick cysteine protease inhibitors with similar activities to these commercial drugs but with additional targets belonging to the psoriasis-related cytokines. However, more profound studies should be conducted about other cytokines as some tested inhibitors promoted their expression.

In summary, the tested tick protease inhibitors, Sialostatin L, Sialostatin L2, Iristatin, and Mialostatin, showed potential therapeutic effects on mannan-induced psoriasis-like skin inflammation through different immune effectors. They are promising candidates for drug development to treat vertebrate immune and inflammatory diseases. While this work proves the potential applicability of tick-derived immunomodulatory proteins in treating psoriasis, further studies are needed to address the specificity of various tick proteins to the central cytokines involved in psoriasis pathogenesis. Importantly, targeting cysteine proteases with specific inhibitors by applying them through the skin might be a good strategy for treating psoriasis with less risk of systemic adverse reactions. In addition, the MISI described herein offers a unique model to study the effect of various inhibitors *in vivo* for future studies with the perspective of understanding/resolving the intriguing pathological cascades of psoriasis.

## Data availability statement

The original contributions presented in the study are included in the article/**Supplementary Materials**, further inquiries can be directed to the corresponding authors.

## Ethics statement

The animal study was approved by the institutional review board of Southern Medical University (l2018183), Guangzhou, China, and performed according to the guidelines of the National Institutes of Health (NIH Publication No. 8023). The study was conducted in accordance with the local legislation and institutional requirements.

## Author contributions

HW: Investigation, Methodology, Writing – original draft, Writing – review & editing. MAJ: Data curation, Formal analysis, Investigation, Methodology, Writing – original draft, Writing – review & editing. JC: Investigation, Writing – original draft. MT: Investigation, Writing – original draft. XX: Data curation, Funding acquisition, Investigation, Supervision, Writing – review & editing. YH: Investigation, Writing – review & editing. KSN: Conceptualization, Formal analysis, Funding acquisition, Investigation, Methodology, Project administration, Supervision, Writing – review & editing. MK: Conceptualization, Data curation, Formal analysis, Funding acquisition, Supervision, Validation, Writing – review & editing.
