# Correction: Tick cysteine protease inhibitors suppress immune responses in mannan-induced psoriasis-like inflammation

**DOI:** 10.3389/fimmu.2025.1699570

**Published:** 2025-09-12

**Authors:** Huimei Wu, Mohamed Amine Jmel, Jinwei Chai, Maolin Tian, Xueqing Xu, Yuan Hui, Kutty Selva Nandakumar, Michail Kotsyfakis

**Affiliations:** ^1^ Guangzhou Medical Research Institute of Infectious Diseases, Department of Pharmacy, Guangzhou Eighth People’s Hospital, Guangzhou Medical University, Guangzhou, China; ^2^ Karolinska Institute United Medical Inflammation Center, School of Pharmaceutical Sciences, Southern Medical University, Guangzhou, China; ^3^ Institute of Parasitology, Biology Centre, Czech Academy of Sciences, České Budějovice, Czechia; ^4^ Guangdong Provincial Key Laboratory of New Drug Screening, School of Pharmaceutical Sciences, Southern Medical University, Guangzhou, China; ^5^ Department of Pulmonary and Critical Care Medicine, Zhujiang Hospital, Southern Medical University, Guangzhou, China; ^6^ Department of Endocrinology, Fifth Affiliated Hospital, Southern Medical University, Guangzhou, China; ^7^ Department of Environmental and Biosciences, School of Business, Innovation and Sustainability, Halmstad University, Halmstad, Sweden; ^8^ Institute of Molecular Biology and Biotechnology, Foundation for Research and Technology-Hellas, Heraklion, Crete, Greece

**Keywords:** autoimmune disease, psoriasis, tick, protease inhibitors, immune responses

Affiliation “Guangzhou Medical Research Institute of Infectious Diseases, Department of Pharmacy, Guangzhou Eighth People’s Hospital, Guangzhou Medical University, Guangzhou, China” was erroneously given as “Department of Pharmacy, The Eighth Affiliated City Hospital of Guangzhou Medical University, The Eighth People’s Hospital of Guangzhou, Guangzhou, China”.

The original version of this article has been updated.

