# Supplementary material for: Tick cysteine protease inhibitors suppress immune responses in mannan-induced psoriasis-like inflammation
Source: Front Immunol. 2024 Feb 20;15:1344878. doi: 10.3389/fimmu.2024.1344878 (PMC10912570; doi:10.3389/fimmu.2024.1344878)
Supplement: Supplementary file 1 [file Table_1.docx]

**Supplementary table 1:**

Table S1. Endotoxin levels in Sialostatin L, Sialostatin L2, Iristatin, and Mialostatin preparations

|  | **Volume, ml** | **Protein, mg/ml** | **Total protein, mg** | **Endotoxin EU/mg** |
| --- | --- | --- | --- | --- |
| **Sialo L** | 8 | 10.75 | 86 | 24.5 |
| **Sialo L Endotoxin free** | 6.2 | 9.64 | 59.8 | 0.023 |
| **Sialo L2** | 6.5 | 12.83 | 83.4 | 15.7 |
| **Sialo L2 endotoxin free** | 5 | 11.09 | 55.5 | 0.035 |
| **Iristatin** | 7 | 1.37 | 29 | 118 |
| **Iristatin endotoxin free** | 10.2 | 1.67 | 24.1 | 0.04 |
| **Mialostatin** | 27 | 4.14 | 19.44 | 35 |
| **Mialostatin endotoxin free** | 8.15 | 2.37 | 14.92 | 3 |

**Supplementary table 2:**

Table S2. Inhibitory potential of Sialostatin L, Sialostatin L2, Iristatin, and Mialostatin against various cysteine proteases as shown by the respective IC50 concentration values expressed in nM.

(n.i not inhibited)

|  | **Sialostatin L** | **Sialostatin L2** | **Iristatin** | **Mialostatin** |
| --- | --- | --- | --- | --- |
| **hCathepsin L** | 1 | 1.1 | 10000 | 1 |
| **hCathepsin S** | 0.2 | 200 | n.i | 2.2 |
| **hCathepsin C** | 670 | 680 | 500 | 2.1 |
| **hCathepsin H** | n.i | n.i | n.i | 29 |
| **hCathepsin B** | n.i | n.i | n.i | 10 |
| **Legumain** | n.i | n.i | n.i | n.i |

**Supplementary table 3:**

Table S3. Amino acid sequences of Sialostatin L, Sialostatin L2, Iristatin, and Mialostatin

| **Cystatin** | **Amino acid sequence** |
| --- | --- |
| Sialostatin L | MTGVFGGYSERANHQANPEFLNLAHYATSTWSAQQPGKTHFDTVAEVVKVETQVVAGTNYRLTLKVAESTCELTSTYNKDTCLPKADAAHRTCTTVVFENLQGDKSVSPFECEAA |
| Sialostatin L2 | MELALRGGYRERSN-QDDPEYLELAHYATSTWSAQQPGKTHFDTVVEVLKVETQTVAGTNYRLTLKVA ESTCELTSTYNKDTCQAN ANAAQRTCTT VIYRNLQGE KSISSFECAAA |
| Iristatin | MFPGVWRKHHPDVDPRYKEWAHFAISSQVENRTNFDTLMTLISVESQVIAGVDYKLKMKVAESTCVIGVDSYSKERCYLKVNVPYMLCTAVVNYMPWEHKTILKSYDCSDRVYGVKSAE |
| Mialostatin | MDICWNSPLFLVCVVLAAAGSASRSKRALVGGWKTQDPTNPKFENLAHYAVSTQVEGREYYDTVLELLEVQTQIVAGVNYKLKFTTTQSTCKIESGVEYSKELCQPKTNKVEAVCTSIIYTVPWQNI KRVLSYHCDA PNNV |

**Supplementary Figure 1: Structure of tick protease inhibitors**


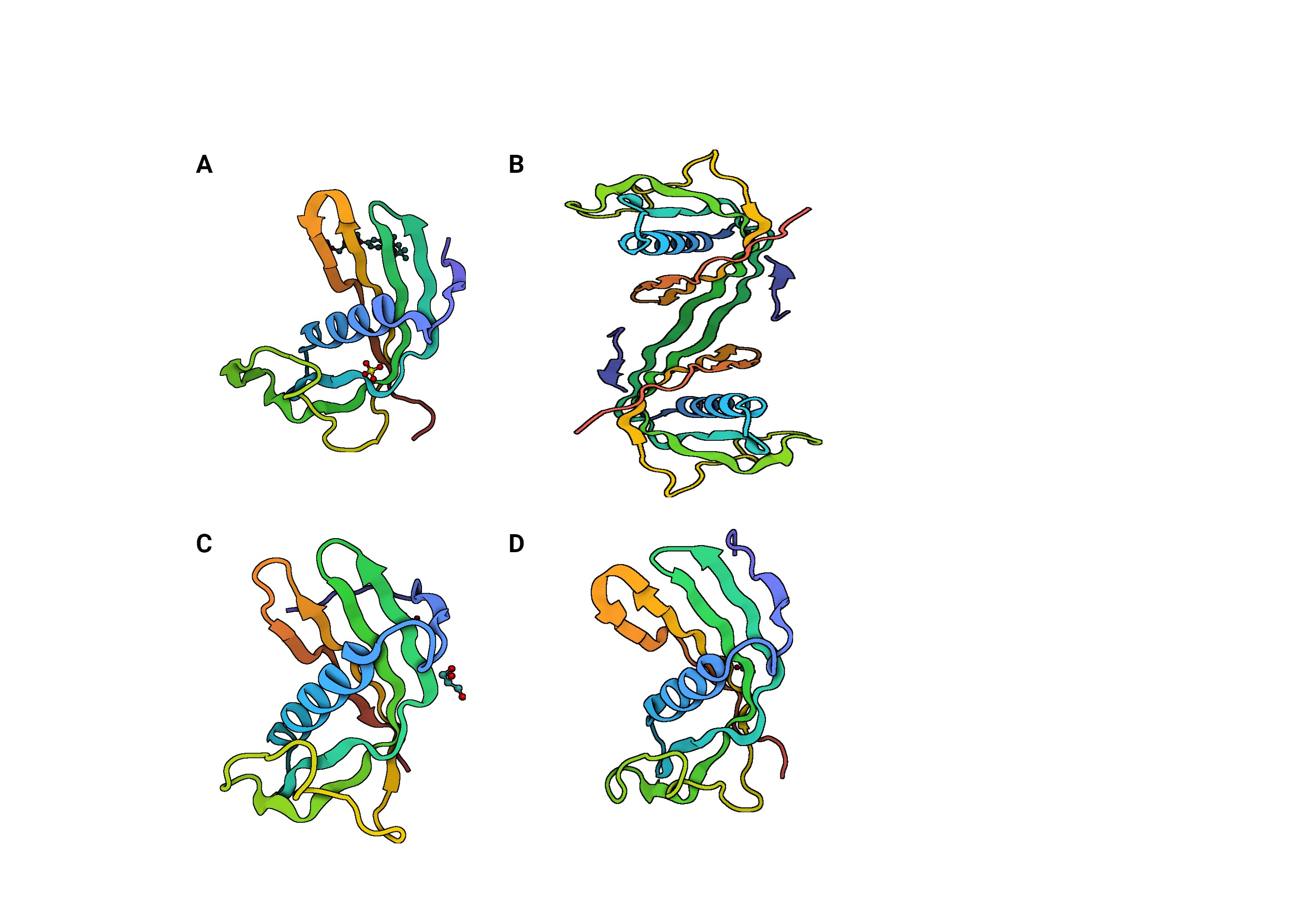


**SF1:** Protein secondary structure of (A) Mialostatin (PDB:6ZTK), (B) Sialostatin L (PDB:4ZM8), (C) Sialostatin L2 (PDB:3LH4) and (D) Iristatin (PDB:5O46).

**Supplementary Figure 2: Toxic effects of tick protease inhibitors on BMDMs.**


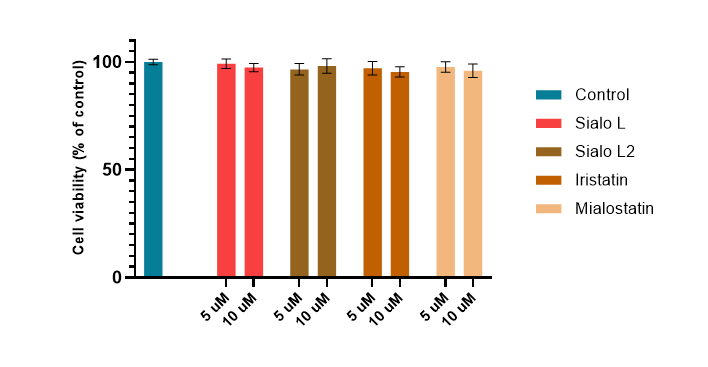


**SF2:** Effect of 5 and 10 µM concentrations of the tested tick protease inhibitors on BMDMs viability. Results were expressed as percentages in comparison to the untreated control group. Sialo L: Sialostatin L; Sialo L2: Sialostatin L2.
